# Dissemination and use of WHO family planning guidance and tools: a qualitative assessment

**DOI:** 10.1186/s12961-018-0321-1

**Published:** 2018-05-22

**Authors:** Joan Marie Kraft, Titilope Oduyebo, Tara C. Jatlaoui, Kathryn M. Curtis, Maura K. Whiteman, Lauren B. Zapata, Mary Eluned Gaffield

**Affiliations:** 1 U.S. Centers for Disease Control and Prevention, Division of Reproductive Health, 4770 Buford Hwy NE, MS F74, Atlanta, GA 30341 United States of America; 2 World Health Organization, Department of Reproductive Health and Research, Avenue Appia 20, 1211 Geneva 27, Switzerland

**Keywords:** Family planning guidance, Research translation, Research use

## Abstract

**Background:**

As countries continue to improve their family planning (FP) programmes, they may draw on WHO’s evidence-based FP guidance and tools (i.e. materials) that support the provision of quality FP services.

**Methods:**

To better understand the use and perceived impact of the materials and ways to strengthen their use by countries, we conducted qualitative interviews with WHO regional advisors, and with stakeholders in Ethiopia and Senegal who use WHO materials.

**Results:**

WHO uses a multi-faceted strategy to directly and indirectly disseminate materials to country-level decision-makers. The materials are used to develop national family planning guidelines, protocols and training curricula. Participants reported that they trust the WHO materials because they are evidence based, and that they adapt materials to the country context (e.g. remove content on methods not available in the country). The main barrier to the use of national materials is resource constraints.

**Conclusions:**

Although the system and processes for dissemination work, improvements might contribute to increased use of the materials. For example, providers may benefit from additional guidance on how to counsel women with characteristics or medical conditions where contraceptive method eligibility criteria do not clearly rule in or rule out a method.

## Background

Low- and middle-income countries (LMICs) continue to improve their family planning (FP) programmes, in line with their national health strategies and commitments to regional (e.g. Ouagadougou Partnership) and global (i.e. Family Planning 2020 (FP2020)) initiatives. Thirty-nine low- and middle-income countries made FP2020 commitments that identify strategies to expand access to high-quality FP services and reach additional users, including improving services and making additional contraceptive methods available [1]. As a set of evidence-based guidance and tools, WHO’s ‘four cornerstones of family planning’ support the provision of high-quality services [2, 3]. We report on analyses of in-depth interviews with maternal and sexual health advisors in five WHO Regional Offices (hereafter WHO regional advisors) and stakeholders in Ethiopia and Senegal to better understand the use of the WHO FP guidance and tools (i.e. materials), their perceived impact and ways to strengthen their use.

WHO’s ‘four cornerstones’ include contraceptive guidance to inform national policy and programme development, as well as resources and tools for providers [4]. Policy-makers and programme managers can use the ‘Medical eligibility criteria for contraceptive use’ (MEC) and the ‘Selected practice recommendations for contraceptive use’ (SPR) booklets to inform national FP guidelines [5, 6]. The MEC provides recommendations for the use of specific contraceptive methods by women and men who have certain medical conditions (e.g. HIV, diabetes) or personal characteristics (e.g. age, parity), and the SPR provides recommendations to safely and effectively provide contraceptive methods (e.g. when a method can be started). ‘Family planning: a global handbook for providers’ (the handbook) is a reference guide or manual containing recommendations from the MEC and SPR, as well as other practice information (e.g. managing side effects) for 20 contraceptive methods [7]. The ‘decision-making tool for family planning clients and providers’ (DMT) is a flip chart that helps providers counsel clients about methods that meet their needs and personal circumstances, while taking into consideration medical eligibility criteria [8]. Assessments of DMT suggest that it improves counselling (e.g. engaged clients, provided tailored information) [9, 10], although evidence for its effects on FP use are mixed [11, 12].

Little is known about how countries access and use these materials. Although WHO does not routinely track the dissemination and use of these materials, some data on their use was collected and published as part of the Strategic Partnership Programme (SPP), a joint initiative of WHO and UNFPA. A report on activities (e.g. workshop, provision of financial and technical support) in Asia-Pacific countries found that countries used the materials in developing or adapting FP policy, establishing standards, improving and conducting training, and developing advocacy and communication materials [3]. Furthermore, a recent baseline assessment of the extent to which sub-Saharan African national FP policies align with WHO guidance on medical eligibility criteria for post-partum FP found that WHO materials were referred to when developing national policies and were used for training [2]. However, neither of these studies described processes for the use of the materials nor how the materials inform providers’ practice, issues that are important to understand the potential benefits of the WHO materials and to identify the need for additional materials or technical assistance.

WHO’s evidence-based materials are intended to bridge the gap between research and practice. Models or frameworks for dissemination of guidance and tools, particularly global guidance, identify the need to adapt materials for the country context, identify and address barriers to use within the country, and describe and monitor implementation strategies [13, 14]. Implementation strategies might include dissemination of materials in the country, training, provision of an adequate stock of materials (e.g. DMT, patient materials), incentives for use, and supportive supervision [14–16]. This descriptive qualitative project sought to answer the following questions: (1) how are WHO materials disseminated; (2) how are they used at the policy (e.g. developing guidelines) and implementation (e.g. use of decision-making tool in FP service settings) levels; and (3) what barriers and facilitators influence the use of WHO materials and materials based in part on WHO materials?

## Methods

Between March and August 2015, three authors (TO, JK, TJ), with backgrounds in family planning programming and evaluation (TO, JK, TJ) service delivery (TO, TJ), and qualitative analysis (JK) conducted in-depth interviews with staff in five WHO regional offices, and stakeholders in Ethiopia and Senegal who are involved in the dissemination and use of the WHO FP materials. In consultation with WHO headquarters staff, we considered several factors when selecting these two countries, including variation in language, perceptions by WHO Regional staff that the countries successfully used the materials, and logistical considerations (e.g. ability to secure interviews with Ministry of Health (MoH) staff and stakeholders). The United States Centers for Disease Control and Prevention determined this project to be public health practice (non-research) and the United States Agency for International Development funded this project.

### Participants

We developed guides and conducted interviews in two phases, starting with five WHO regional advisors who provide technical assistance for sexual and reproductive health, including FP in African Region, South-East Asia Region, European Region, Eastern Mediterranean Region and Western Pacific Region (Table 1). For our purposive sample of WHO regional advisors, we selected participants in consultation with WHO headquarters staff who provided input into the protocol for the project. We contacted six potential participants by e-mail from March to June 2015, introduced the interviewer (TO), provided information about the project (i.e. research questions, nature of interviews), and asked them to participate and identify a time for the interview. Five of the six people contacted agreed to participate. One of the authors (TO) conducted all of these interviews. Interviews were audio-recorded and transcribed. Questions and prompts in the interview guide focused on how WHO disseminates the materials, perceptions of how the materials are used by countries, and countries that have used the materials with variable levels of success (e.g. integrated new methods into their method mix on the basis of WHO materials and evidence) (to inform the selection of countries). Interviews lasted approximately 1 hour and we did not compensate participants for their time.

**Table 1:** Interview participants and topics

Dates	Participants	Interview Topics
March – June 2015	WHO Regional Advisors (maternal and sexual health) (*n* = 5): African Region, South-East Asia Region, European Region, Eastern Mediterranean Region and Western Pacific Region	• Dissemination strategies and tracking • How materials are used by countries • Barriers and facilitators to use • Countries using the materials successfully
July – August 2015	Stakeholders in Ethiopia and Senegal • Policy-makers (formulate national FP policy and guidelines) (*n* = 6) • Disseminators (disseminate and support use of materials) (*n* = 7) • Implementers (support use of and use materials in their organisations) (*n* = 4) • End-users (physicians, nurses, other FP providers) (*n* = 4)	• Access to WHO materials • Use of WHO materials • Barriers and facilitators to use • Training and dissemination of national materials (based on WHO materials) • Perceived impact of WHO materials

Based on input from the first phase, we selected Ethiopia and Senegal for the second phase. From July to August 2015, three authors (TO, JK, TJ) conducted interviews with four groups of stakeholders, namely policy-makers (i.e. formulate national policy and guidelines), disseminators (i.e. disseminate and support use of materials), implementers (i.e. use materials in their own services and support use by public services), and end-users (i.e. physicians, nurses or other FP service providers). Recognising that complex factors shape the use of WHO materials, we developed separate interview guides based on each group’s role in support for and use of the materials. We interviewed more policy-makers (*n* = 6 interviews) and disseminators (*n* = 7 interviews) than implementers (*n* = 4 interviews) and end-users (*n* = 4 interviews) in both countries to better understand whether and how countries use the WHO materials, how they disseminate adapted or original WHO materials in-country, and how they support the use of national materials.

For our purposive sample, we used an iterative process to identify participants. WHO staff helped us identify a staff member from the MoH that directed or was deeply involved in development of FP guidelines, a staff member from each WHO country office, and implementing partners (i.e. professional associations and non-government organisations (NGOs) that work in FP). When we contacted MoH and WHO country office staff to inform them about the project and request their participation, we asked for names of other organisations to include in the project. Only one potential participant in Ethiopia did not respond to our request for an interview. Most participants had over 10 years of experience in FP and had worked in more than one FP position during their careers, often working in the public sector and NGOs at some point.

We contacted all participants by e-mail (and phone if they did not respond to e-mail) to introduce the interviewers, explain the project (i.e. research questions, nature of the interview), request their participation, and set up interviews. With one exception, two authors conducted in-person interviews (TO conducted one phone interview). One author (TO) led the interviews, and another author took notes and asked clarifying questions (JK in Ethiopia, TJ in Senegal). The participation of two authors for interviews allowed us to address saturation in real-time; as the interviewers noted recurrent descriptions of some topics (e.g. use of WHO materials for revising national FP guidelines), they discussed saturation and decided whether to reduce focus on those topics in future interviews within a country.

Interviews were conducted in participants’ offices and, in a few cases (*n* = 5), more than one person from an organisation participated in an interview. Interviews in Ethiopia were conducted in English. Interviews in Senegal were conducted with the assistance of a translator who translated from English to French and vice versa, if needed. With one exception, interviews were audio-recorded and transcribed (we relied on notes for the exception). Interviews lasted approximately 1 hour and participants were not compensated for their time. Questions and prompts in the interview guides focused on how participants learn about WHO materials, how they use WHO materials for developing national guidelines and policies, whether and to what extent WHO materials are used in service delivery, and how nationally developed and WHO materials are disseminated and used.

### Analysis

For this qualitative descriptive analysis, one author (JK) read and coded segments of text (from two sentences to one page) in word processing documents, starting with codes based on the research questions (e.g. dissemination strategies, guideline development, use of WHO materials in FP programmes) and adding codes as they emerged from her reading of the transcripts. The additional codes provided context for the use of WHO materials (i.e. MoH structure, FP goals and objectives, and other FP initiatives). Given the descriptive nature of the project, JK reviewed segments of text for each code to summarise processes for using and adapting the WHO materials, dissemination and use, as well as to identify barriers and facilitators and perceived impact of the WHO materials. Other authors reviewed and commented on initial summaries, drawing on written notes from interviews and/or their experiences participating in the interviews. Although we did not verify transcripts with participants, we solicited participants’ feedback on the report and did not receive substantive feedback.

## Results

### Country context

Ethiopia and Senegal have relatively high rates of unmet need for contraception (25.2% in Senegal in 2015 and 22.3% in Ethiopia in 2016) [17, 18]. Both countries have made ambitious commitments to increase access to high-quality FP services to increase contraceptive use (i.e. as part of FP2020, in 2012 Ethiopia committed to reaching a contraceptive prevalence rate (CPR) of 69% by 2015 and Senegal committed to a CPR of 29% by 2015) [19, 20]. Both countries developed strategies to improve the quality of FP services and to generate and increase the demand for and use of contraception. Participants from Ethiopia and Senegal noted that implementing partners work on promoting task shifting to community health workers (CHWs), increasing access to long-acting reversible contraception, post-partum and post-abortion FP counselling and provision, HIV/FP integration, social franchising, reducing stock-outs, demand generation, and measurement and evaluation. One implementing partner usually pilots an improvement and assists in national roll out. Further, some participants mentioned that the evidence base in WHO materials was drawn upon for these efforts.

### Dissemination of WHO materials

Collectively, the WHO regional advisors described a multi-faceted dissemination strategy, starting with translation into a few key languages (e.g. French, Spanish), and working with partners, particularly UNFPA, for dissemination. Regional advisors reported that WHO uses a number of channels for dissemination, including WHO collaborating centres, regional or country-specific launch or technical meetings, side events at international meetings, professional organisations (e.g. “*congress of gynaecologists and obstetricians or midwives*”), dissemination of “*soft copies and paper copies…to our colleagues at country level and also with partners*”, and newsletters and listserves. Through these channels, WHO hopes to reach country stakeholders (e.g. MoH, FP programme implementers) directly (e.g. regional workshops) and indirectly (e.g. professional organisations). Participants noted that in-person dissemination events allow them to address questions and concerns (e.g. about long-acting and reversible contraception or contraception for adolescents) and give participants a chance to begin planning their use of the materials. Due to costs, distribution of printed copies at these events is usually limited to one per participant. Requests for additional copies are possible; however, more than 50 or 100 copies typically need to be purchased.

Other than one regional advisor who mentioned the SPP (see Background), participants did not describe systematic or long-term efforts to support the use of materials.

“…*and this process* [the SPP] *was conducted in a systematic way. So every year we meet --- we introduce new guidelines…we support country activities in the viewing, adapting and adopting these guidelines….Unfortunately…the first trial was around…three years. And by the year 2004 the project stopped*.” (Regional advisor, 1)

Further, almost all WHO regional advisors reported that they did not systematically track the dissemination of WHO materials.

“*There are no mechanisms that I can press a button and say 63 member states will answer this question*.” (Regional advisor, 2)

One regional advisor noted that periodic surveys and special assessments sometimes provide insight into use of the materials.

Participants from Ethiopia and Senegal identified similar channels for accessing WHO materials, namely learning about materials from staff at WHO country offices, meetings and the internet. According to participants, WHO Country Offices share information and updates with the MoH and the FP technical working group (TWG) to prompt revisions and updates. For example, in Ethiopia, the WHO country office may write a concept paper to the MoH, suggesting changes based on the updated MEC. In Senegal, the WHO country office has held workshops to share materials. However, one participant in Senegal noted that communication with WHO was not consistent, and would like additional information about some recommendations in the MEC. When implementing partners hear about WHO updates (e.g. at meetings, through professional networks, and from listserves), they share information with the TWG and suggest updates.

### Use of WHO materials for developing and adapting national materials

WHO regional advisors reported that countries use the materials to inform national policies, strategies and guidelines. For example, two regional advisors reported that countries integrate WHO recommendations into their national FP guidelines (e.g. have integrated best practices documented in WHO materials, have added new methods to the method mix in the country or removed restrictions on use of methods based on non-medical criteria, use WHO materials, such as the DMT, in service delivery).

Participants from Ethiopia and Senegal echoed the responses from the WHO regional advisors, and provided more information about how they use the WHO materials. Both countries used WHO materials in their ongoing efforts to improve access to and the quality of services. The structure and processes for using materials was similar in both countries. The MoH takes a leadership role in all matters, working with regional and district health officials, implementing partners, donors and other Ministries:

“…[MoH] *coordinates National FP…providing technical guidance, coordination and leadership to increase or improve the FP programme*.” (Ethiopia participant)

In both countries, the MoH convenes a TWG, comprised of implementing partners, regional and district health officers, and donors, to revise national guidelines (or protocols) and standardised training materials every 5 to 7 years and updates them in the interim when information (e.g. update to MEC) becomes available. The TWG:

“*…has a responsibility for programme improvement* [in] *family planning. So we are discussing if there is* [a need for] *a guidelines revision, for instance you know the WHO Cornerstones are available so the new information, evidence-based information is coming,… especially the medical eligibility criteria is changed…and the training also needs to* [be] *revised.*” (Ethiopia participant).

Once a decision is made to revise or update national guidelines and training materials, a smaller group, led by an implementing partner with relevant expertise, makes revisions and solicits feedback from the larger TWG (and ultimately the Minister of Health). During this process, both countries rely heavily, although not exclusively, on the MEC and SPR:

“…*we only use the WHO…the other organisations* [e.g. International Planned Parenthood Federation] *use the same guidelines as WHO. And we haven’t seen many differences*.” (Senegal participant)

Participants in both countries trust the WHO materials and reported that evidence is critical; further, they reported that, if they use other materials, they have to be evidence-based and provide explanations for changes, like in the WHO materials. In part, this is because they believe that evidence is needed to persuade policy-makers and providers to make changes.

WHO regional advisors and participants from Ethiopia and Senegal reported that countries consider their own context when deciding how to incorporate some of the WHO recommendations into their policy and training materials. For example, two WHO regional advisors noted that it took time to make emergency contraception (EC) available in their regions because of the concern that young girls might access EC. The countries went through a process to understand and get buy-in to adopt EC. Participants in Ethiopia and Senegal elaborated on this theme; when the countries make adaptations, it is largely for cultural issues. This does not mean that they rule out the use of particular methods (e.g. EC, intrauterine device (IUD)) by a particular group. In fact, participants in both countries reported that the updated information in the MEC on IUDs led them to change their guidance to allow for their use by adolescents and adapt messaging to address cultural realities:

“*…we have to adapt the ideas to our culture. When we talk with young people about family planning, our first concept is abstinence*.” (Senegal participant)

Participants in Ethiopia reported adapting the DMT for their CHWs (known as health extension workers). Ethiopia simplified the DMT to reflect the methods health extension workers provide (condom, pill, injectable) and their level of education and training (i.e. more pictures, fewer words, translated into Amharic). Otherwise, participants in both countries reported that they distribute the WHO handbook, DMT and a MEC chart, without revision, to providers during training.

### Rolling out training and WHO materials for providers

To standardise services, the MoH in each country expects all FP providers (public, NGO, private) to follow the guidelines, and to use training and other materials that the TWG adapts/develops. In addition, the MoH expects providers to have and use the handbook, the DMT and a MEC summary chart, as well as client materials they develop (e.g. brochure, posters). Participants from NGOs that provide services (i.e. International Planned Parenthood Federation, Marie Stopes International) discussed the importance of adhering to the national standards:

“*…we always encourage them* [our staff] *to use the national one* [guideline, training] *since we are governed by the national literatures and policies*.” (Ethiopia participant)

Implementing partners play a large role in rolling out revised and updated guidelines, training materials and WHO materials (i.e. handbook, DMT and MEC chart). In consultation with the MoH, partners in each country agree to work with regional and district health offices to provide support to public health facilities in one or more regions (and sometimes to specific districts within regions):

“*….our staff* [is] *in the service of the Ministry of Health…*.[we are] *aligned to the national health plan and we … support the Ministry of Health to achieve its national objectives… we developed our action plan in collaboration with … the government*.” (Senegal participant)

Working with the MoH and regional health offices, partners provide support for training (using the national training materials), tools and materials for providers (including WHO materials), capacity-building, technical assistance, supportive supervision and sometimes equipment.

“*Our support includes, building the capacity of the health facilities. That means providing training to health workers… provided as per the national standard curriculum and then we provide them with the necessary material equipment … ongoing support to the facilities, like we do regular supportive supervision, technical support to the providers and also other necessary support to the facility as a whole. We also engage the management system.*” (Ethiopia participant)

Training (and refresher trainings) offers routine opportunities for increasing provider awareness of the revised guidelines and training on WHO materials to be used in clinical practice, including the handbook, the DMT, a summary chart of the MEC, the pregnancy checklist (for ruling out pregnancy in order to start contraceptive methods), and various client materials (e.g. poster, examples of methods). Both countries use a ‘train the trainers’ approach, training master trainers who train others to train facility-level providers. In both countries, training includes ‘theoretical’ (sharing of information) and ‘practical’ (role play, observation of counselling and provision with real clients) components, using the national (i.e. standardised) training materials and PowerPoint slides (mentioned in Ethiopia only).

“*When leading training, they are in two sessions; there is the theoretical training, which is sometimes for certain methods … and practical training. And during this part, demonstrations are given … And, for example, if we are talking about an IUD or an implant, we use insertion kits and removal kits*.” (Senegal participant)

Different training modules are used, depending on the needs and skill of the providers (e.g. postpartum FP modules used when training antenatal care providers to deliver anticipatory FP counselling). Although materials for the training (except for CHW) are in English (Ethiopia) or French (Senegal), the trainers use local languages during trainings to facilitate understanding.

Both countries have built-in efforts to facilitate the use of national and WHO materials and ensure that providers have the skills to deliver quality services and use the materials provided during training. Knowledge checks and observation checklists (during practical training) are used to help ensure that trainees develop knowledge and skills, including counselling skills. In addition, the MoH in Ethiopia has asked each implementing partner to participate in an evaluation to help improve and ensure quality training. The MoH in each country organises supervisory visits to a select number of facilities during the year (quarterly or twice yearly). During the visits, teams made up of staff from the MoH and partners check up on the use of materials, as well as other aspects of care. In Ethiopia, the team reviews a number of services, so there is less time to focus on FP.

Participants in both countries reported barriers to skills building (through training and supportive supervision) and providers’ use of WHO materials. Funding and resource constraints are significant; neither country has sufficient funds or partners to offer training, supportive supervision and materials to all public facilities. In Ethiopia, participants wanted to use the MEC wheel (rather than the chart), but noted that it was too expensive to buy from WHO and there was no company in Ethiopia that had the materials and equipment to produce the wheel locally (using an electronic file from WHO). Participants in Senegal noted that they do not always have sufficient materials to distribute to providers during training sessions. In addition, staff turnover is high and there are no mechanisms for tracking whether a new provider in a facility received training and materials in a previous position, or where staff go when they leave one facility. A few participants in Ethiopia said that this resulted in inefficient use of training resources (e.g. some providers are trained twice, others are not trained at all). Finally, participants in Ethiopia noted that, although they liked the DMT, providers were not always counselling on all methods due to a lack of time. One participant mentioned an evaluation that found that providers themselves indicated that they did not always counsel on all methods.

### Perceptions of WHO materials

We asked participants in Ethiopia and Senegal to identify changes and improvements in their FP programmes in the past 5 years, as well as the role of the WHO materials in those changes. In both countries, participants cited statistics showing increased contraceptive use and mentioned programmatic efforts that led to those changes, including efforts to reduce stock-outs (e.g. ‘informed push’ model in Senegal), task shifting to community health workers, and demand generation activities (e.g. working with Imams in Senegal). Participants reported that the standardisation of materials, based on the WHO evidence-based materials, improved FP service provision, including counselling and provision of a wider range of methods (e.g. the updated MEC contributed to reduced provider biases around IUDs).

Participants also had questions or concerns with particular WHO tools. In both countries, some participants noted that the wording (i.e. double negative) of the pregnancy checklist was a problem, particularly for staff who spoke English/French as a second language. Although participants said providers understand the four MEC categories and the associated colouring on the MEC summary chart, providers had some difficulty differentiating and operationalising categories 2 and 3. Concerns and difficulties stemmed from providers’ desire to ensure that women receive an appropriate method of their choice on that day.

“*You have to put the woman on the safe side. We don’t want to give them a kind of exploration or experiment*.” (Ethiopia participant).

Further, in Ethiopia, providers at one health centre were concerned that additional testing to determine if a woman met the eligibility criteria for a method might mean that she would leave without a method, and might not return to the facility for a contraceptive method.

Although it was not mentioned by participants from Ethiopia or Senegal, based on their experiences working with countries and other partners, WHO regional advisors noted gaps in the materials, including the need for more information on managing side effects, materials for training, and more information on how to start women on a particular method.

“*…it would be good to emphasise how to handle the side effects of contraceptives, because sometimes it is not easy for providers. They have a lot of questions*.” (Regional advisor, 3)

## Discussion

In summary, respondents reported on the use of a multi-faceted strategy to disseminate WHO’s evidence-based FP materials. Both WHO regional advisors and participants from Senegal and Ethiopia reported that the materials are used in the revision of guidelines and development of training materials. In addition, Ethiopia and Senegal offer copies of WHO materials (i.e. DMT, handbook, MEC chart, pregnancy checklist) to providers during training, and expect them to use them in practice. To the extent possible within funding constraints, providers are trained using standardised training materials that are informed by WHO materials. Given the constraints, it is unclear whether supportive supervision visits provide an opportunity for further improvement in services. Although the participants have some questions about certain WHO materials, they trust WHO and its materials and believe that the materials, along with a number of initiatives (e.g. task shifting, post-partum FP, reduced stock-outs, expansion of demand-generation efforts) have contributed to improved FP services and increased FP use in their countries.

This project is not without limitations. It is difficult to generalise from qualitative data, particularly when data were collected in two countries that were thought to have some success in their use of the WHO materials and that have some resources (e.g. donor-funding) and political will for FP programming. In addition, we focused on initial dissemination from WHO to countries and how countries use the WHO materials to revise their national guidelines, materials and training. Although we touched on how providers use the national materials, we did not explore changes in provider behaviour/services in detail. More information is needed to understand how providers receive and use their country’s revised materials. However, given the scarcity of this information in the published and grey literature, it is important to understand the processes countries use when adapting the WHO materials to their needs and to identify where additional information or support might be needed as a baseline that can potentially lead to improved dissemination and use.

Indeed, our analysis identified areas for improvement throughout the process. The multi-faceted dissemination strategy directly and indirectly reaches MoH and implementing partners in both countries. However, the sometimes limited interaction between WHO and countries can leave country-level decision-makers with questions and a desire for more consistent communication with WHO. In particular, enhanced communication when updates are released and when TWGs are revising/updating guidelines and materials might be particularly important, as countries want to know and understand the rationale for why changes are made. It may be beneficial to build on existing efforts (e.g. tracking dissemination and use of SPR 3rd edition) to identify standard indicators for dissemination, use and barriers to use, systematically track dissemination and uptake, and identify the most effective dissemination channels and potential need for changes in dissemination and/or the WHO materials.

Stakeholders in Ethiopia and Senegal described strong systems to incorporate updated WHO materials into their guidelines, training and other materials (e.g. TWGs review updated materials, agree on need for change, and charge a sub-group to make revisions). However, their description of provider difficulties using some materials suggests the need to make more adjustments than for cultural issues alone. For example, with regard to the MEC, country policy-makers and programme planners might consider issues in their countries that make it more difficult for providers to determine which category of recommendation is the most reasonable and provide guidance so that healthcare providers know how to handle situations common to country context (e.g. what to do if testing would delay provision of a method). Further, modifying the wording of tools, such as the pregnancy checklist, might make the tools easier to use.

Other barriers to the use of the WHO materials, and to quality FP service delivery, stem from resource, staffing and time constraints. Those barriers may require a revision in service delivery. For example, participants generally liked the DMT and expected providers to use it. Given time constraints, however, policy-makers and programme planners might think of alternatives. They might consider developing patient education and decision-making materials, or having a peer counsellor work with a client before the client sees a provider.

Many of these changes are best made at the country level, with detailed information about the local context. National policy-makers and programme planners may benefit from and require technical assistance from WHO at these times. That said, WHO regional advisors (but not country stakeholders) noted the need for more information on managing side effects, given the role that these, and other misperceptions, play in non-use and discontinuation [21, 22]. Developing guidance on the management of side effects, with input from countries on the nature of side effects reported and how they are currently trying to manage them, might improve the uptake and use of the MEC.

## Conclusions

Our project describes how two countries use WHO’s evidence-based ‘four cornerstones of family planning’. In addition to other initiatives, stakeholders reported that the materials contributed to improved FP services. Although the system and processes for dissemination are working, improvements in the system might contribute to increased use of the materials. Brief assessments in countries that are not successfully using the materials might identify other ways to improve the system, as would assessments of how providers receive and use country-specific materials.

## Abbreviations

CHW: community health worker
DMT: Decision-making Tool for Family Planning Clients and Providers
EC: emergency contraception
FP: family planning
FP2020: Family Planning 2020
IUD: intrauterine device
MEC: Medical Eligibility Criteria for Contraceptive Use
MoH: Ministry of Health
SPP: Strategic Partnership Programme
SPR: Selected Practice Recommendations for Contraceptive Use
TWG: technical working group
